# Hard Sample Mining for GNSS Spoofing Detector via Physics-Based Meta-Learning Optimization

**DOI:** 10.3390/s26216849

**Published:** 2026-10-29

**Authors:** Liupeng Chen, Gang Ou, Jing Yu, Weihua Mou

**Affiliations:** 1 College of Electronic Science and Technology, National University of Defense Technology, Changsha 410073, China; chenliupeng213@163.com (L.C.); ougangcs@139.com (G.O.); yjisee@163.com (J.Y.); 2 National Key Laboratory for Positioning, Navigation and Timing Technology, Changsha 410073, China

**Keywords:** GNSS, spoofing detection, hard sample mining, signal quality monitoring, moving variance, meta-learning

## Abstract

GNSS spoofing detectors flag spoofing signals through sensing features such as carrier-to-noise density ratio and signal quality monitoring statistics. Training stronger detectors—and mapping detector performance boundaries—requires hard traction samples that systematically challenge those features, yet such samples are expensive to obtain under closed-loop replay and vary with the traction target. We propose Physics-Based Meta-Learning Optimization (PBMO) as a hard sample miner: it archives traction trajectories across traction targets, warm-starts a new traction target with Offline-RGPE and physics-based priors, and refines candidates with Physics-prior Guided Pattern Search (PhGPS). Hardness is scored by a conformal upper bound on the fraction of moving-variance windows that exceed channel thresholds. On a four-channel moving-variance (MV) detector, comparisons show that PBMO lowers the mean conformal upper bound by 30.7% relative to an unoptimized frequency-difference-lock strategy, and under the same online search budget remains below cold-start, nearest-neighbor, and mean-trajectory initializations. A USRP X310 wired loopback at one traction frequency preserves this ordering.

## 1. Introduction

GNSS provides positioning, navigation, and timing for aerospace and ground platforms, yet open civil signals remain vulnerable to generated spoofing. Consequently, receivers increasingly deploy spoofing detectors—carrier-to-noise density ratio ($C/N_{0}$), signal quality monitoring (SQM), moving-variance (MV) features, eye-diagram or measurement-consistency checks, and learning-based detectors—to flag anomalous authentic–spoofing [1,2,3,4,5,6]. Strengthening these detectors through hard-example or curriculum learning, and mapping their performance boundaries under standardized stimuli, both require hard samples: traction trajectories that systematically suppress detection while still achieving loop pull-off [7,8]. Because traction schedules are time-varying and detection probability $P_{d}$ has no closed form, such samples must be mined through repeated instrumented closed-loop runs rather than inferred from signal design alone.

Hard sample mining under asynchronous traction faces two practical constraints. First, the traction target sets maneuver duration and feasible power/frequency trajectories; a cold start at a new traction target can consume most of a fixed evaluation budget before the pull-off requirement is met. Second, receiver noise makes each replay stochastic; ranking trajectories from one trial or a sample mean can mislabel hardness and offers no finite-sample guarantee for comparison. We, therefore, use conformal prediction to form a distribution-free upper bound $P_{d,\mathrm{upper}}$ on the window-exceedance fraction $P_{d}$ [9,10], and treat that bound as a conservative hardness score: under the same pull-off constraint, lower $P_{d,\mathrm{upper}}$ indicates a harder sample for the detector under test. Section 3.3.2 gives the exchangeability assumption, separates the calibration replicates from the runs that score a new candidate, and states the finite-sample level used with the replicate counts of this study.

Meta-learning and transfer methods can cut early evaluations by recycling past optima [11,12], but most of that literature targets AutoML-scale hyperparameters with statistical meta-features. Hard sample mining at a new traction target is different: the decision vector is a high-dimensional, target-dependent traction trajectory, and cross-target similarity is physical—traction-target distance and four-channel MV response vectors. We propose Physics-Based Meta-Learning Optimization (PBMO) as a hard sample miner under a fixed online evaluation budget. It builds a cross-target archive of mined trajectories, warm-starts new traction targets with Offline-RGPE and physics-based priors, and refines candidates with Physics-prior Guided Pattern Search (PhGPS). The empirical study instantiates this miner on a four-channel MV detector and verifies selected schedules with a wired loopback.

The contributions of this paper are:

- A conformal upper bound $P_{d,\mathrm{upper}}$ on detection probability, used as a conservative hardness score (lower is harder), with calibration residuals separated from the runs that score a new candidate and with the finite-sample coverage level stated for the replicate counts used here.
- A PBMO hard sample miner that reweights archived traction trajectories by ranking, traction-target proximity, and four-channel MV response-vector similarity, then applies physics-guided corrections and PhGPS refinement at a new traction target under a fixed online evaluation budget. The warm start transfers archived trajectories by an offline product of those three scores.
- A SoftSim–SoftGNSS case study on a four-channel MV detector, with a USRP X310 wired loopback check at one traction frequency, that reports hardness scores, equal-online-budget comparisons, and the separate offline costs.

The rest of the paper is organized as follows. Section 2 reviews related work. Section 3 defines the hardness score and the hard sample mining problem. Section 4 describes PBMO. Section 5 presents the sensing setup and results. Section 6 and Section 7 discuss implications and conclude.

## 2. Related Work

### 2.1. GNSS Asynchronous Traction Stimuli for Hard Sample Generation

Field experiments have shown that civil GPS users can be steered off course by generated spoofing rather than by brute-force jamming [13,14]. When asynchronous traction is adopted as a generative stimulus, a central modeling question is how power and Doppler schedules move the false code into the tracking loop without an abrupt break in lock. Peng et al. [15] studied that hand-off as an alignment-then-pull process. Much of the follow-on work still starts from closed-form power and Doppler profiles: Gao and co-authors stabilize the correlator by holding a controlled carrier-frequency offset [7,16], and have also formulated asynchronous traction within distributed forwarding-spoofing deployments [17]. Other authors reduce the power needed for capture through multipath-assisted cancellation [18], or redesign the traction code itself so that SQM statistics on BPSK look quieter [19], though the latter can disturb code–carrier consistency. Song et al. [20] take a different cut at the same problem: instead of designing a stealthier signal, they bound how quickly an asynchronous attack may advance before a static receiver’s code loop fails to stay locked, and they relate that limit to ordinary loop knobs such as order, bandwidth, and damping. However, they do not mine alternative traction schedules that maximize detector difficulty under a fixed number of instrumented receiver evaluations—the gap addressed by our hard sample mining workflow.

### 2.2. GNSS Anti-Spoofing Sensing and Detector Stress Testing

Prior work has established sensing workflows for quantifying GNSS integrity observables rather than only classifying spoofing after the fact. Soft-receiver and multi-correlator chains measure Q-channel and SQM features under controlled IF replay [2,3]; correlation-domain eye-diagram detectors further stress peak-shape exposure beyond classical Ratio/Delta tests [5] and measurement-domain consistency checks (e.g., pseudo-range difference/sum linearity) complement correlator-level detectors at the navigation layer [21]. Recent surveys systematize these jamming/spoofing detectors across receiver stages [4]. Song et al. [20] further treat asynchronous traction as an instrumented stress test and measure an admissible pull-in rate bound for static receivers—a physical loop-locking rate bound tied to ordinary loop knobs. Physics-informed learning detectors that fuse residual GNSS observables with optimized classifiers [6] emphasize physics-guided features for binary classification; they still need a supply of hard traction samples for hard-example or curriculum learning. The miner developed here supplies such samples for a four-channel MV detector; curriculum use of those samples is left as a prospective application.

Across these works, what remains underdeveloped is hard sample mining when the traction stimulus itself is a high-dimensional, traction-target-dependent schedule and hardness must be scored conservatively under stochastic SoftGNSS replay. This paper addresses that gap with a SoftSim–SoftGNSS–USRP instrument chain, a conformal upper bound $P_{d,\mathrm{upper}}$ as the hardness score, and PBMO to accelerate search for hard traction trajectories—complementing pull-in-rate studies [20] with a hard-sample generation procedure for a four-channel MV detector.

### 2.3. Meta-Learning Optimization and Cross-Task Transfer

Outside GNSS, expensive black-box search often borrows strength from previous tasks. Ranking-weighted GP ensembles (RGPE) fit one Gaussian process per source task and reweight those surrogates on a new task by a ranking loss [11]; other lines pretrain priors across mismatched domains, meta-learn acquisition rules without an explicit likelihood [22], or harden meta-BO against outlier tasks [23]. Those methods grew up around AutoML workloads with comparatively cheap queries. Here each query is a full SoftGNSS pass, the online evaluation cap is small, and similar tasks are nearby traction targets with comparable four-channel MV response vectors. Under those constraints, the warm start blends archived hard schedules and retunes them with physics-based channel cues.

## 3. Problem Formulation

For clarity, the scenario without a spoofing signal is called the clean scenario. Asynchronous traction is a standard generative stimulus for GNSS anti-spoofing hard sample mining: the stimulus signal gradually advances its code phase—while keeping code and carrier consistent—until the receiver loop follows the false signal [20,24]. The mining problem is to find feasible traction trajectories that achieve a commanded Doppler pull-off while minimizing a conservative hardness score built from detection probability $P_{d}$ (and its conformal upper bound).

### 3.1. Asynchronous Traction Spoofing Signal Model

A spoofing signal is a delayed replica of the authentic signal with identical navigation bits and PRN code; power, code delay, and carrier phase differ. Exact carrier-phase alignment would require centimeter-level knowledge of the receiver antenna, so the traction schedule typically controls carrier Doppler instead.

In the instrumented sensing chain, SoftSim synthesizes a matching spoofing component while the receiver loop remains locked on the authentic signal. Phase and power are adjusted so that sliding code phase gradually captures the tracker without breaking lock [25]. Three parameter-locking schemes are common. The frequency-unlock strategy preserves the fixed code-to-carrier Doppler ratio of the GNSS signal:

(1) $$F=\frac{f_{\mathrm{code}}}{f_{\mathrm{carr}}}=\frac{R_{c}}{f_{L}}$$

where $f_{\mathrm{code}}$ and $f_{\mathrm{carr}}$ are code and carrier Doppler, $R_{c}$ is the chip rate, and $f_{L}$ is the carrier frequency.

Although this scheme passes consistency tests, large carrier offsets can break lock and induce correlator-amplitude swings detectable in power.

**The frequency-lock strategy** keeps carrier Doppler matched while raising code Doppler to capture the loop; correlator amplitude stays stable, but the code–carrier ratio drifts and triggers consistency alarms.

**The frequency-difference-lock strategy** fixes the authentic–spoofing carrier offset while holding its rate of change to zero, which stabilizes correlator amplitude [7]. Code Doppler follows from consistency constraints. Because only correlator behavior is modeled, reference schedules set this way need not maximize detector difficulty—motivating the hardness scores and mining objective developed below.

### 3.2. Decision Variable

Primary mining parameters are code phase, carrier Doppler, and power. Code and carrier Doppler are tied by a fixed ratio, so only one frequency coordinate needs to be varied in search. The miner searches residuals ΔP and ΔF on top of a frequency-difference-lock reference trajectory.

Traction unfolds over time. Following [26], the local code must close to within 1.5 chips of the authentic code before the discriminator is dominated by the spoofing signal. Minimum maneuver duration follows from code Doppler separation and initial code offset:

(2) $$T=\frac{\Delta \tau +1.5}{f_{\mathrm{code}}^{s}-f_{\mathrm{code}}^{a}}$$

where Δτ is the initial code offset (the spoofing typically lags the authentic signal) and $f_{\mathrm{code}}^{s}$, $f_{\mathrm{code}}^{a}$ are the spoofing and authentic code Doppler.

To shrink the search space, the maneuver is split into *K* segments; segment-duration ratios enter the decision vector alongside power and frequency residuals.

The decision vector is, therefore,

(3) $$x=\left[\Delta P^{s},\;\Delta F^{s},\;r\right]$$

where $\Delta P^{s}$, $\Delta F^{s}$, and r are power residuals, frequency residuals, and segment-duration ratios.

### 3.3. Hardness Score from Detection Probability

In hard sample mining, sample difficulty relative to the detector under test is measured by the window-exceedance fraction $P_{d}$ under a prescribed traction stimulus: lower $P_{d}$ means fewer MV windows cross a threshold, hence a harder sample for this monitor. The definition below is the four-channel MV statistic used throughout the study.

#### 3.3.1. Spoofing Detection Probability

$C/N_{0}$ tracks received power relative to noise density and is among the most accessible observables [4,27,28]. While the loop tracks the authentic signal, the spoofing appears as extra noise and depresses $C/N_{0}$; as power rises and lock transfers, $C/N_{0}$ recovers. Sustained monitoring of its drift and, therefore, the flags spoofing.

SQM metrics compare correlator shape when authentic and spoofing signals interact [3,5,8]. Common tests include Ratio, Delta, and early–late phase (ELP); spoofing distorts the correlation peak and drives these statistics outside nominal bands.

Further, moving variance (MV) can smooth short-lived fluctuations and yields a more stable test statistic [1,29]:

(4) $$\begin{array}{cc} MV(n) & =\frac{1}{w}\sum_{i=(n-1)(w-1)+1}^{(n-1)(w-1)+w}a^{2}\left(i\right)\\ \; & \;-{\left(\frac{1}{w}\sum_{i=(n-1)(w-1)+1}^{(n-1)(w-1)+w}a\left(i\right)\right)}^{2},\\ \; & \;n=1,2,\ldots ,N \end{array}$$

where a(i) is the *i*-th metric sample, *w* is the sliding window length, and *N* is the number of windows. Typically, MV values spike in selected windows after spoofing begins. Following [1], the detection threshold follows from

(5) $$G\left(\frac{w-1}{2},\frac{\left(w-1\right)T_{h}}{2M}\right)=1-P_{fa}$$

where $T_{h}$ is the detection threshold, G(·,·) is the regularized gamma function, $P_{fa}$ is the false-alarm probability, and *M* is the mean MV under clean scenarios.

Because traction is time-varying, $P_{d}$ cannot be derived in closed form; it is estimated as the fraction of MV windows that exceed channel thresholds:

(6) $$\begin{array}{cc} P_{d} & =\frac{1}{N}\;\mathrm{Num}\{\left(MV_{\mathrm{CNo}}\left(n\right)>T_{C}\right)\;\vee \;\left(MV_{\mathrm{Ratio}}\left(n\right)>T_{R}\right)\\ \; & \;\vee \;\left(MV_{\mathrm{Delta}}\left(n\right)>T_{D}\right)\;\vee \;\left(MV_{\mathrm{ELP}}\left(n\right)>T_{E}\right)\} \end{array}$$

where $T_{C}$, $T_{R}$, $T_{D}$, and $T_{E}$ are MV thresholds on the $C/N_{0}$, Ratio, Delta, and ELP channels; Num{·} counts qualifying windows. Higher $P_{d}$ indicates easier samples for the detector; lower $P_{d}$ identifies harder samples.

Equation (6) is the fraction of MV windows, inside one closed-loop trial, that exceed at least one channel threshold. All hardness scores, bounds, and comparisons below use this window-exceedance fraction. Because this fraction fluctuates across noise realizations, ranking trajectories by one trial or by a sample mean can favor lucky draws. The next section replaces that point estimate by a conformal upper bound and states the finite-sample coverage level under which the bound is used.

#### 3.3.2. Conformal Prediction

We use split conformal prediction [9,10]. With exchangeable calibration residuals of simulated $P_{d}$, a (1−α) quantile margin *q* gives the upper endpoint

(7) $$P_{d,\mathrm{upper}}\left(X_{n+1}\right)=\hat{\mu }\left(X_{n+1}\right)+q$$

where $\hat{\mu }$ is a local repeated-run mean,

(8) $$\begin{array}{cc} \ell & =\min\;\left(1,\;\frac{\left\lceil (n+1)(1-\alpha )\right\rceil }{n}\right),\\ q & ={\mathrm{Quantile}}_{\ell }\left({\left\{S_{i}\right\}}_{i=1}^{n}\right) \end{array}$$

and $S_{i}=\left|Y_{i}-\hat{\mu }\left(X_{i}\right)\right|$. The finite-sample guarantee requires that the nonconformity scores used to form *q* and the score of a newly drawn SoftGNSS replicate be exchangeable, i.e., that they arise from the same receiver and noise process so that their joint distribution is invariant to permutation. Under that assumption, with $\alpha =\alpha_{\mathrm{cp}}$ in (8),

$$P\left(S_{\mathrm{new}}\leq q\right)\geq 1-\alpha_{\mathrm{cp}},$$

and for the absolute margin likewise $P\left(P_{d}^{\mathrm{new}}\leq \hat{\mu }+q\right)\geq 1-\alpha_{\mathrm{cp}}$. The probability is marginal over the calibration pool and the new score.

For spoofing schedules, $P_{d}$ is observed only through repeated runs, so the residual pool is built from those repeats. Let $S_{\mathrm{cal}}={\left\{x_{j}\right\}}_{j=1}^{J}$. Strategy $x_{j}$ is run $R_{j}$ times with local mean and spread

(9) $$\begin{array}{cc} {\hat{\mu }}_{j} & =\frac{1}{R_{j}}\sum_{r=1}^{R_{j}}P_{d}^{\left(j,r\right)}\\ {\hat{\sigma }}_{j} & =\max\;\left(\sigma_{\min},\;\sqrt{\frac{1}{R_{j}-1}\sum_{r=1}^{R_{j}}{\left(P_{d}^{\left(j,r\right)}-{\hat{\mu }}_{j}\right)}^{2}}\right) \end{array}$$

where $\sigma_{\min}>0$ avoids near-zero denominators. Absolute and normalized residuals are

(10) $$\begin{array}{cc} e_{j,r}^{\mathrm{abs}} & =|P_{d}^{\left(j,r\right)}-{\hat{\mu }}_{j}|\\ e_{j,r}^{\mathrm{norm}} & =\frac{e_{j,r}^{\mathrm{abs}}}{{\hat{\sigma }}_{j}} \end{array}$$

Pooling over $S_{\mathrm{cal}}$ and using the level *ℓ* in (8) gives the margins

(11) $$\begin{array}{cc} q_{\mathrm{abs}} & ={\mathrm{Quantile}}_{\ell }\left(\{e_{j,r}^{\mathrm{abs}}\}\right)\\ q_{\mathrm{norm}} & ={\mathrm{Quantile}}_{\ell }\left(\{e_{j,r}^{\mathrm{norm}}\}\right) \end{array}$$

In this study, the calibration set $S_{\mathrm{cal}}$ consists solely of the multi-replicate labeling runs on the nine archived training trajectories: after pattern search at each training frequency, the retained schedule is re-evaluated R=5 times, residuals (10) are formed, and all residuals are pooled (n=45). The resulting $q_{\mathrm{abs}}$ and $q_{\mathrm{norm}}$ are computed once at $\alpha_{\mathrm{cp}}=0.15$ (so ℓ=40/45≈0.889) and then frozen. Single-run evaluations used as the search objective during pattern search are never entered into this pool.

For a new strategy x with *R* independent runs,

(12) $$\begin{array}{cc} \hat{\mu }\left(x\right) & =\frac{1}{R}\sum_{r=1}^{R}P_{d}^{\left(r\right)}\left(x\right)\\ \hat{\sigma }\left(x\right) & =\max\;\left(\sigma_{\min},\;\mathrm{std}\left(\{P_{d}^{\left(r\right)}\left(x\right)\}\right)\right) \end{array}$$

these *R* runs are drawn after calibration has been closed. They provide only the local mean and spread that enter (13); they are not appended to the residual pool and do not change $q_{\mathrm{abs}}$ or $q_{\mathrm{norm}}$. The dual bounds

(13) $$\begin{array}{cc} P_{d,\mathrm{upper}}^{\mathrm{abs}}\left(x\right) & =\hat{\mu }\left(x\right)+q_{\mathrm{abs}}\\ P_{d,\mathrm{upper}}^{\mathrm{norm}}\left(x\right) & =\hat{\mu }\left(x\right)+q_{\mathrm{norm}}\;\hat{\sigma }\left(x\right) \end{array}$$

cover low-spread and high-spread cases, respectively. We take

(14) $$P_{d,\mathrm{upper}}\left(x\right)=\max\;\left(P_{d,\mathrm{upper}}^{\mathrm{abs}}\left(x\right),\;P_{d,\mathrm{upper}}^{\mathrm{norm}}\left(x\right)\right)$$

as the hardness score used below. The pointwise maximum is the more conservative of the two endpoints and is used as a ranking score at the nominal marginal level $1-\alpha_{\mathrm{cp}}=0.85$.

### 3.4. Optimization Objective

The hard sample mining objective is to identify feasible traction trajectories with the lowest conformal upper bound on the window-exceedance fraction among those achieving a successful pull-off—i.e., the hardest samples for this MV monitor within the online evaluation budget. We take pull-off success as the hard feasibility constraint and minimize the conformal upper bound $P_{d,\mathrm{upper}}$ on that basis.

Pull-off succeeds when the traction schedule captures the tracking loop and reported Doppler deviates from the clean scenarios. Given traction target $f_{t}$, success requires code and carrier offsets to reach $f_{t}$ and remain stable. Because code and carrier Doppler have different units, deviations are compared through proportionality factor *F* from Equation (1):

(15) $$\mathrm{Err}={\left(\Delta f_{\mathrm{code}}-f_{t}\right)}^{2}+{\left(\Delta f_{\mathrm{carr}}/F-f_{t}\right)}^{2}$$

where $\Delta f_{\mathrm{code}}$ and $\Delta f_{\mathrm{carr}}$ are the realized pull-offs. Both $\Delta f_{\mathrm{code}}$ and $\Delta f_{\mathrm{carr}}/F$ are in hertz: *F* in Equation (1) maps carrier Doppler onto the code-Doppler scale, and $f_{t}$ is the commanded code-Doppler target. Err is a sum of squared Doppler errors with units ${\mathrm{Hz}}^{2}$, and $\tau =10^{-4}\;{\mathrm{Hz}}^{2}$ is the pull-off feasibility tolerance. Trajectories with Err>τ are treated as infeasible. The hard sample mining problem is

(16) $$\min_{x\in X}\;P_{d,\mathrm{upper}}\left(x\right)\;s.t.\;\mathrm{Err}\left(x\right)\leq \tau .$$

## 4. Method

### 4.1. Overall Architecture

Hard sample mining couples three difficulties: multiple traction frequencies, high-dimensional continuous traction trajectories, and expensive, noisy black-box receiver replay. When $f_{t}$ changes, so do maneuver length and the feasible power/frequency profile. PBMO addresses these difficulties as a hard sample miner through three phases: training builds cross-frequency hard-sample archives; warm start transfers historical trajectories via Offline-RGPE and physics-based priors; refinement executes PhGPS local search from the warm-start iterate.

**Figure 1:**
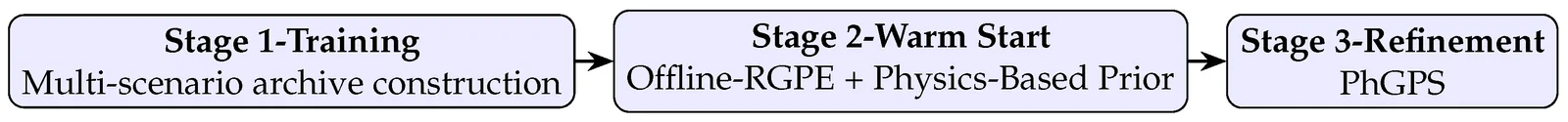
Overall PBMO hard sample mining framework.

Figure 1 outlines PBMO. The training phase runs multi-start PS on training frequencies ${\left\{f_{t}^{i}\right\}}_{i=1}^{N}$, archives conformally calibrated hard samples $x_{i}^{*}$ with hardness scores $P_{d,\mathrm{upper}}^{\left(i\right)}$, and precomputes Offline-RGPE weights and physics-based prior sensitivity templates. At a new $f_{t}^{\mathrm{new}}$, the warm-start phase forms $x_{\mathrm{rgpe}}$ via Offline-RGPE reweighting, evaluates it once to obtain four-channel MV response vector $v={\left[v_{C},v_{R},v_{D},v_{E}\right]}^{⊤}$, and applies physics-based MV-channel priors to obtain $x_{\mathrm{init}}$. The refinement phase runs PhGPS local search from $x_{\mathrm{init}}$ to produce a mined hard sample $x^{*}$.

The remainder of this section follows the three PBMO phases. Section 4.2 details multi-scenario archive construction; Section 4.3 and Section 4.4 detail warm start via Offline-RGPE and physics-based priors; Section 4.5 describes PhGPS refinement.

### 4.2. Multi-Scenario Archive Construction

The training phase runs once before mining at new frequencies. It populates scenario-indexed hard-sample archives consumed by warm start and refinement: verified trajectories $\left\{x_{i}^{*}\right\}$, conformal hardness scores $P_{d,\mathrm{upper}}^{\left(i\right)}$, four-channel MV response vectors $v_{i}$, and global conformal margins $\left(q_{\mathrm{abs}},q_{\mathrm{norm}}\right)$.

Multi-Start Pattern Search on Training Frequencies. For each training frequency $f_{t}^{i}\in F_{\mathrm{train}}$, multi-start PS launches $n_{\mathrm{start}}$ independent searches from perturbed zero or random iterates projected onto X. To stretch the budget, search minimizes single-run ${\hat{P}}_{d}\left(x\right)$ while enforcing Err(x)≤τ through a uniform infeasibility penalty. Across *N* scenarios, this yields a diverse set of feasible, hard (low-$P_{d}$) trajectories rather than a single frequency-specific optimum.

Multi-Replicate Re-Evaluation and Archive Labeling. After PS completes at $f_{t}^{i}$, the retained candidate is re-evaluated with *R* independent simulations, the conformal hardness score $P_{d,\mathrm{upper}}^{\left(i\right)}$ is formed via Equations (12)–(14), and the four-channel MV response vector $v_{i}={\left[v_{C},v_{R},v_{D},v_{E}\right]}_{i}^{⊤}$ is recorded. This vector supports four-channel MV response-vector similarity (Equation (19)) and physics-based prior validation.

Pooled Conformal Calibration. Training pools residuals from all calibration replicates across scenarios to estimate global margins $q_{\mathrm{abs}}$ and $q_{\mathrm{norm}}$ (Equations (10) and (11)) at significance level $\alpha_{\mathrm{cp}}$. These margins stabilize $P_{d,\mathrm{upper}}\left(\cdot \right)$ under the limited replicate counts used at multi-replicate re-evaluation and during refinement, so final candidate ranking relies on a consistent conservative hardness score rather than point estimates of $P_{d}$.

### 4.3. Offline-RGPE Dynamic Scenario Weighting

The warm-start phase begins by reweighting archived training strategies for a new target frequency. The central difficulty is scenario dependence: when $f_{t}^{\mathrm{new}}$ changes, so do $T_{\mathrm{dyn}}$, pull-off rate, and the best schedule of ΔP and ΔF. A cold start, therefore, burns budget before Err≤τ. Conventional RGPE [11] fits a Gaussian process per source task and reweights those surrogates from target observations; related transfer methods likewise update from target feedback [12]. Offline-RGPE retains only the ranking-weighted transfer idea—prefer historically strong archives and avoid re-evaluating all *N* of them on the new task—but forms weights offline, without source-task GPs or target-driven weight updates, and transfers the archived trajectories $x_{i}^{*}$ themselves.

Conformally scored strategies ${\left\{x_{i}^{*}\right\}}_{i=1}^{N}$ from training frequencies are blended by relevance to $f_{t}^{\mathrm{new}}$ and projected to the feasible set. Relevance is the product of the ranking scores, frequency proximity, and four-channel MV response-vector similarity defined next; the physics-based correction in Section 4.4 and PhGPS poll ordering follow this blend.

#### 4.3.1. Offline Ranking Score

Hard samples with similar four-channel MV response vectors at different $f_{t}$ often share ramp shapes. The ranking score, therefore, up-weights archives with low conformal hardness scores $P_{d,\mathrm{upper}}^{\left(i\right)}$:

(17) $$s_{i}^{\mathrm{rank}}=\exp\;\left(-\frac{\ell_{i}}{\tau_{r}\cdot \mathrm{med}\left(\left\{\ell_{j}\right\}\right)}\right)$$

where $\ell_{i}=P_{d,\mathrm{upper}}^{\left(i\right)}$ is the calibrated upper bound for training scenario *i*; $\mathrm{med}(\left\{\ell_{j}\right\})$ normalizes scale across frequencies and temperature $\tau_{r}$ controls how sharply weight concentrates on the best archives.

#### 4.3.2. Frequency Proximity

Under frequency-difference-lock traction, $f_{t}^{\mathrm{new}}$ sets the target pull-off and couples ΔF through consistency. Equation (2) links maneuver duration to code Doppler, so nearby training frequencies yield similar ramp profiles. An inverse-distance kernel captures this continuity:

(18) $$s_{i}^{\mathrm{freq}}=\frac{|f_{t}^{i}-f_{t}^{\mathrm{new}}{|}^{-1}}{\sum_{j}{\left|f_{t}^{j}-f_{t}^{\mathrm{new}}\right|}^{-1}}$$

#### 4.3.3. Four-Channel MV Response-Vector Similarity

Traction-target proximity ignores strategy-shape differences at the same $f_{t}$. The four-channel MV response vector $v_{i}={\left[v_{C},v_{R},v_{D},v_{E}\right]}_{i}^{⊤}$ summarizes how each MV metric contributes to detection and captures parameter–detector coupling. Cosine similarity against the training mean $\bar{v}$ favors structurally aligned archives:

(19) $$s_{i}^{\mathrm{ch}}=\max\;\left(0,\;\frac{v_{i}^{⊤}\bar{v}}{∥v_{i}∥\;∥\bar{v}∥}\right)$$

where $v_{i}$ records the exceedance rates of the four MV metrics in that scenario.

#### 4.3.4. Combined Weights

The three scores are multiplied—with response-vector similarity mapped to [0.5,1] so noisy vectors cannot zero out a weight—and normalized:

(20) $$\begin{array}{cc} {\tilde{w}}_{i} & =s_{i}^{\mathrm{rank}}\cdot s_{i}^{\mathrm{freq}}\cdot \left(0.5+0.5s_{i}^{\mathrm{ch}}\right)\\ w_{i} & =\frac{{\tilde{w}}_{i}}{\sum_{j}{\tilde{w}}_{j}} \end{array}$$

The affine channel term $0.5+0.5s_{i}^{\mathrm{ch}}$ retains half the baseline weight when similarity is low, improving robustness to noisy $v_{i}$.

The warm-start strategy is the weighted combination

(21) $$x_{\mathrm{rgpe}}=\;\sum_{i=1}^{N}w_{i}\;x_{i}^{*}$$

### 4.4. Physics-Based Prior

Warm start continues by applying physics-based MV-channel priors to refine $x_{\mathrm{rgpe}}$ along detector-informed directions. The traction schedule takes over the loop by adjusting segment-wise power and frequency residuals ΔP and ΔF. If perturbations to ΔP and ΔF can be linked to individual MV channels, the search can decide whether to adjust power or frequency first. From Equation (6), $P_{d}$ fires when any MV metric exceeds its threshold; the four-channel MV response vector v, whose entries record exceedance rates on $C/N_{0}$, Ratio, Delta, and ELP, localizes the dominant triggering metric for each scenario.

As is well known, $C/N_{0}$ responds mainly to power (ΔP), whereas Ratio, Delta, and ELP respond more strongly to frequency (ΔF) [28]. When v shows elevated $C/N_{0}$ exceedance, corrections push on ΔP; when Ratio, Delta, and ELP dominate, corrections push on ΔF. Because the mapping is scenario dependent, sensitivities are estimated to verify whether the aforementioned prior mapping aligns with experimental results by local perturbation experiments around baseline $x_{0}$ rather than by closed-form models.

On nine training frequencies $f_{t}\in \left[0.050,0.0525,\ldots ,0.070\right]$ Hz, with baseline $x_{0}$ as the neighborhood center, univariate uniform offsets are applied separately to ΔP and ΔF: ΔP∈[−1.5,1.5] and $\Delta F\in [-0.15f_{t},0.15f_{t}]$ each at 11 grid points, with offsets applied to all *K* time segments; each grid point is evaluated 10 times and four-channel MV response vectors v are recorded.

For scenario *i*, numerical gradients of v are computed on the ΔP (or ΔF) grid, absolute values are taken and averaged across grid points, yielding

(22) $$\begin{array}{cc} S_{\Delta P}^{\left(i\right)} & =E\left[|\partial v/\partial \Delta P|\right]\\ S_{\Delta F}^{\left(i\right)} & =E\left[|\partial v/\partial \Delta F|\right] \end{array}$$

**Figure 2:**
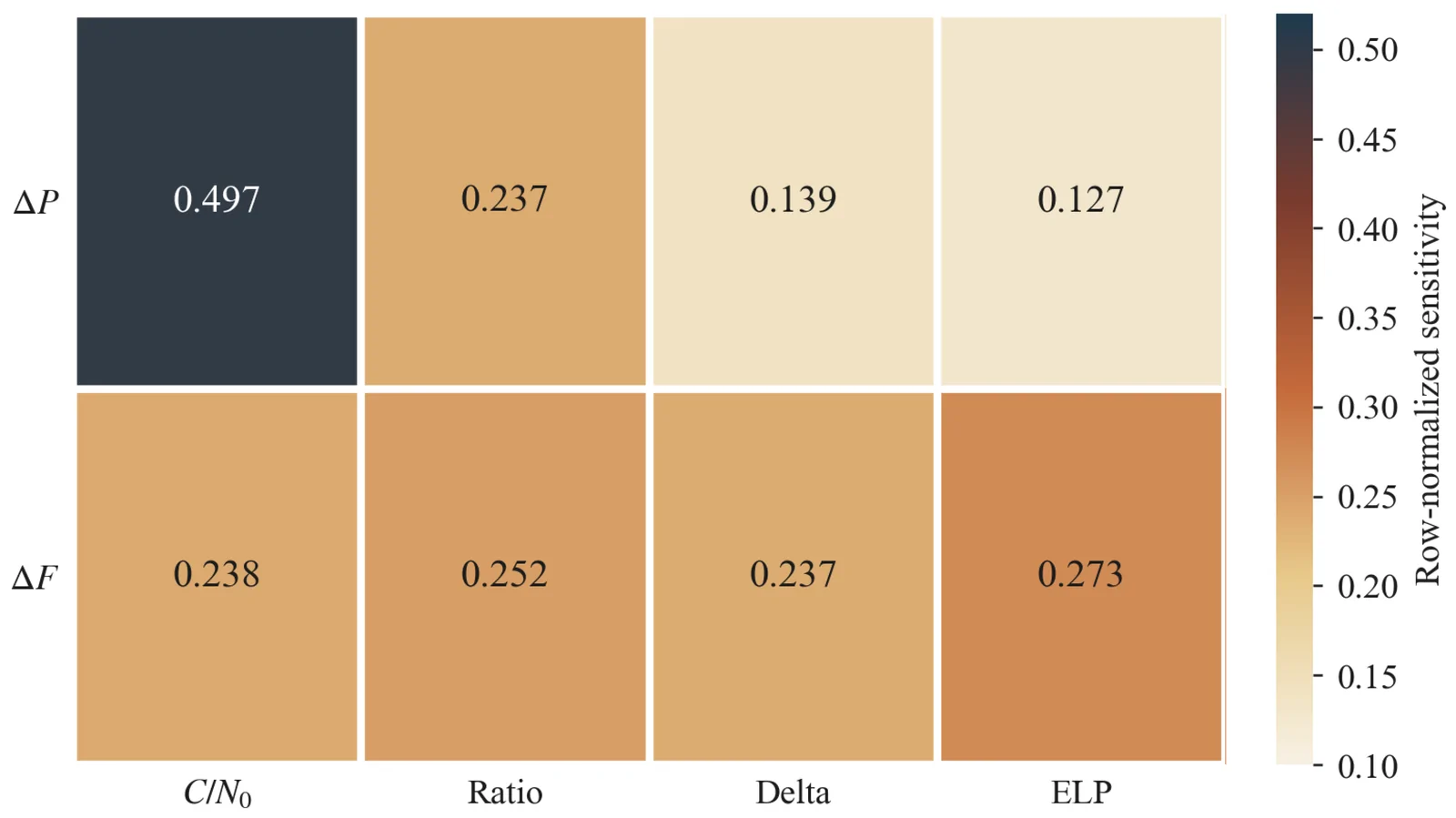
Normalized heatmap $\bar{S}$ of four MV channels under ΔP and ΔF perturbations.

Arithmetic averaging and normalizing across all training scenarios yields mean channel sensitivity $\bar{S}=\left[{\bar{S}}_{\Delta P}^{⊤};{\bar{S}}_{\Delta F}^{⊤}\right]$, as shown in Figure 2.

Figure 2 shows that ΔP perturbations affect the $C/N_{0}$ channel most strongly, with Ratio second and Delta/ELP weaker; ΔF perturbations raise all four channels by comparable amounts, with ELP slightly highest. Further, $C/N_{0}$ and Ratio account for 73% of ΔP sensitivity, whereas Ratio, $C/N_{0}$, and ELP account for 76% of ΔF sensitivity. Elevated $C/N_{0}$/Ratio exceedance, therefore, calls for ΔP corrections; elevated Ratio/Delta/ELP exceedance calls for ΔF corrections. The measured four-channel MV response vector v is mapped to search biases along the two residual chains:

(23) $$\begin{array}{cc} dP_{\mathrm{bias}} & \propto {\hat{v}}_{C}+w_{R}^{P}\;{\hat{v}}_{R}\\ dF_{\mathrm{bias}} & \propto {\hat{v}}_{E}+w_{R}^{F}\;{\hat{v}}_{R}+w_{D}^{F}\;{\hat{v}}_{D} \end{array}$$

where the subscripts *C*, *R*, *D*, and *E* denote the $C/N_{0}$, Ratio, Delta, and ELP MV channels, respectively. The weights are $w_{R}^{P}={\bar{S}}_{\Delta P,R}/{\bar{S}}_{\Delta P,C}\approx 0.48$, $w_{R}^{F}={\bar{S}}_{\Delta F,R}/{\bar{S}}_{\Delta F,E}\approx 0.92$, and $w_{D}^{F}={\bar{S}}_{\Delta F,D}/{\bar{S}}_{\Delta F,E}\approx 0.87$.

To validate the mapping, the power-side target is $G_{P}^{1}=\left\{C/N_{0},\mathrm{Ratio}\right\}$ and the frequency-side target is $G_{F}^{1}=\left\{\mathrm{Ratio},\mathrm{Delta},\mathrm{ELP}\right\}$. Their controls are $G_{P}^{2}=\left\{\mathrm{Delta},\mathrm{ELP}\right\}$ and $G_{F}^{2}=\left\{C/N_{0}\right\}$. A correct prior should yield higher weighted sensitivity on the target group. Validation ratios are

(24) $$\begin{array}{cc} \rho_{P} & =\frac{{\bar{S}}_{\Delta P,C}+w_{R}^{P}{\bar{S}}_{\Delta P,R}}{{\bar{S}}_{\Delta P,D}+{\bar{S}}_{\Delta P,E}}\\ \rho_{F} & =\frac{w_{R}^{F}{\bar{S}}_{\Delta F,R}+w_{D}^{F}{\bar{S}}_{\Delta F,D}+{\bar{S}}_{\Delta F,E}}{{\bar{S}}_{\Delta F,C}} \end{array}$$

Larger $\rho_{P}$ and $\rho_{F}$ indicate stronger alignment between the correction direction specified by the prior and the measured coupling.

**Figure 3:**
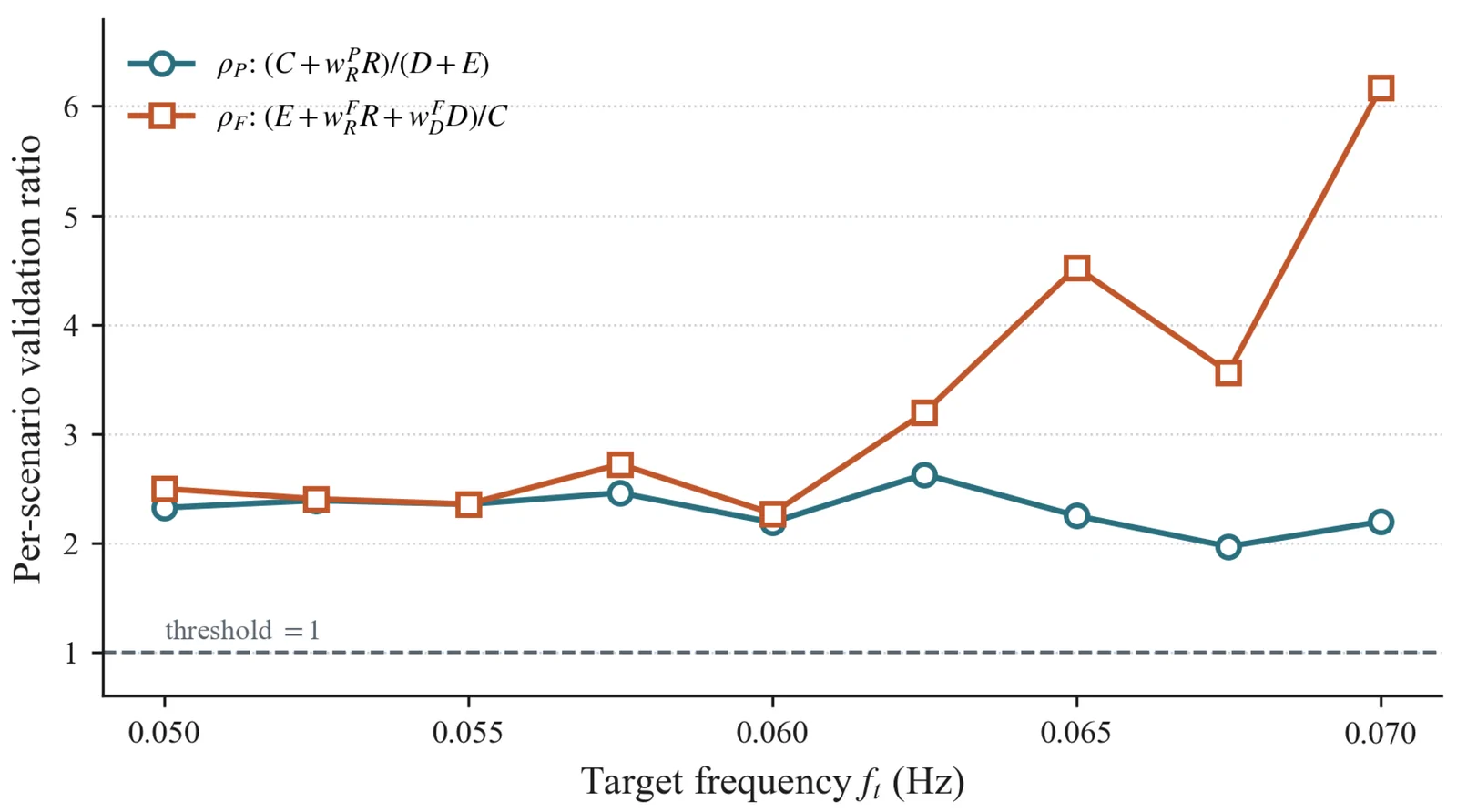
Per-scenario validation ratios as a function of $f_{t}$.

Figure 3 shows $\rho_{P}>1$ and $\rho_{F}>1$ at all nine training frequencies, with nine-scenario averages of 2.29 and 2.98 and minima of 1.97 and 2.27. The channel ordering underlying the prior does not invert within the training band.

A correction vector $\delta_{\mathrm{ch}}$ is built from the $\ell_{1}$-normalized four-channel MV response vector $\tilde{v}$ and prior gain γ:

(25) $$\begin{array}{cc} dF_{\mathrm{bias}} & =\gamma \left({\tilde{v}}_{E}+w_{R}^{F}{\tilde{v}}_{R}+w_{D}^{F}{\tilde{v}}_{D}\right)\\ dP_{\mathrm{bias}} & =\gamma \left({\tilde{v}}_{C}+w_{R}^{P}{\tilde{v}}_{R}\right) \end{array}$$

where $dF_{\mathrm{bias}}$ and $dP_{\mathrm{bias}}$ bias the search toward frequency and power residuals, respectively.

These biases are applied uniformly across all *K* segments:

(26) $$\begin{array}{cc} \delta_{F,k} & =\eta_{F}\;dF_{\mathrm{bias}}\\ \delta_{P,k} & =\eta_{P}\;dP_{\mathrm{bias}},\;k=1,\ldots ,K \end{array}$$

Segment corrections are stacked into the physics-based vector

(27) $$\delta_{\mathrm{ch}}\left(v\right)=\left[\eta_{P}\;dP_{\mathrm{bias}}\;1_{K};\;\eta_{F}\;dF_{\mathrm{bias}}\;1_{K};\;0\right]$$

where 0 leaves temporal proportions unchanged; $\eta_{F}=0.006$ and $\eta_{P}=0.12$ balance correction magnitudes across units.

Corrections may violate physical validity, so the warm-start iterate is obtained by projection:

(28) $$x_{\mathrm{init}}=\Pi_{X}\;\left(x_{\mathrm{rgpe}}+\delta_{\mathrm{ch}}\left(v\right)\right)$$

where $\Pi_{X}\left(\cdot \right)$ clips ΔP and ΔF to physical bounds and normalizes temporal proportions so that $\sum_{k}r_{k}+r_{\mathrm{tail}}=1$.

### 4.5. Physics-Prior Guided Pattern Search

Warm start places the iterate near a good basin but does not guarantee a minimal hardness score. The refinement phase, therefore, runs budget-limited PS around $x_{\mathrm{init}}$ in two passes: a coarse mesh with a cheap single-run objective, then a fine mesh minimizing $P_{d,\mathrm{upper}}$. Coordinate polling alone can spend evaluations on dimensions weakly coupled to the detector channels, eroding the benefit of warm start.

The physics-based MV-channel priors from Section 4.4 guide not only initialization but also poll ordering. Poll steps follow the same perturbation–response logic as the MV chain, and likely descent directions are tried first. Under the same budget, this ordering accelerates feasibility recovery and subsequent improvement.

#### 4.5.1. Robust Objective and Two-Stage Fitness

Let $P_{d}^{\left(m\right)}\left(x\right)$ denote the detection probability from the *m*-th independent evaluation, with sample mean $\hat{\mu }\left(x\right)$ and sample standard deviation $\hat{\sigma }\left(x\right)$. The feasible set is defined as

(29) F={x∈X:Err(x)≤τ},

Infeasible candidates receive a uniform large penalty, yielding gated fitness

(30) $$\Psi \left(x;J\right)=\left\{\begin{array}{cc} J(x), & x\in F,\\ \Phi (x), & x\notin F \end{array}\right.$$

where Φ(x) penalizes infeasible points and J(x) is the objective on F.

The coarse PS pass minimizes a single-run surrogate for rapid exploration—the same fast objective used during training search:

(31) $$J_{\mathrm{fast}}\left(x\right)={\hat{P}}_{d}\left(x\right)$$

where ${\hat{P}}_{d}\left(x\right)$ is the detection rate from one simulation.

The fine PS pass re-evaluates each candidate *R* times and minimizes the conformal upper bound:

(32) $$J_{\mathrm{robust}}\left(x\right)=P_{d,\mathrm{upper}}\left(x\right)$$

#### 4.5.2. Physics-Prior Guided Pattern Search (PhGPS)

Pattern search polls coordinate directions around $x_{k}$: if fitness improves, the iterate moves; otherwise the mesh $\Delta_{k}$ contracts and polling continues until the stage budget is exhausted. With poll directions $D=\{d_{1},\ldots ,d_{p}\}$, candidates are

(33) $$P_{k}=\left\{x_{k}+\Delta_{k}\;d_{\ell }:\;\ell =1,\ldots ,p\right\}$$

If $\min_{z\in P_{k}\cap X}\Psi \left(z;J\right)<\Psi \left(x_{k};J\right)$, the best candidate becomes $x_{k+1}$ and polling restarts; otherwise $\Delta_{k+1}<\Delta_{k}$ and polling continues at $x_{k}$. Each candidate is projected through $\Pi_{X}\left(\cdot \right)$ to enforce temporal and bound constraints. Standard PS polls $\pm e_{j}$ in fixed index order; PhGPS instead ranks dimensions by search bias from Equation (25). After $\ell_{1}$-normalizing v, poll priority for dimension *j* is

(34) $$s_{j}=\left\{\begin{array}{cc} |dP_{\mathrm{bias}}|, & j\in \{1,\ldots ,K\}\;\\ |dF_{\mathrm{bias}}|, & j\in \{K+1,\ldots ,2K\}\;\\ s_{\min}, & j\in \{2K+1,\ldots ,n\}\; \end{array}\right.$$

where $s_{\min}=0.1\min(|dP_{\mathrm{bias}}|,|dF_{\mathrm{bias}}\left|\right)$ keeps temporal dimensions from being ignored. Larger $s_{j}$ means dimension *j* is more likely to relieve the currently dominant triggering metric.

Each poll round sorts dimensions by s:

(35) $$\begin{array}{cc} \pi & =\arg\mathrm{sort}(s)\\ s_{\pi (1)} & \geq s_{\pi (2)}\geq \cdots \geq s_{\pi (n)} \end{array}$$

where π reorders coordinates by descending $s_{j}$, unlike fixed-index PS.

At iterate $x_{k}$ with mesh $\Delta_{k}$, each coordinate π(j) generates two projected candidates:

(36) $$\begin{array}{cc} z_{j}^{\left(+\right)} & =\Pi_{X}\;\left(x_{k}+\Delta_{k}e_{\pi (j)}\right)\\ z_{j}^{\left(-\right)} & =\Pi_{X}\;\left(x_{k}-\Delta_{k}e_{\pi (j)}\right) \end{array}$$

where $e_{\pi (j)}$ is the unit vector along dimension π(j).

A first-improvement rule accepts the first descent direction found, then recenters polling at $x_{k+1}$. This avoids evaluating all 2n directions before moving.

If no direction improves fitness, the poll fails, $x_{k}$ is retained, and the mesh contracts to $\Delta_{k+1}=\rho \;\Delta_{k}$ until tolerance or budget is reached.

PhGPS and standard PS share the same candidate set and mesh update; only the poll order differs. Under tight budgets, trying exceedance-sensitive coordinates first often determines whether search enters the feasible region before evaluations run out.

The refinement phase alternates coarse and fine PS passes:

(37) $$\begin{array}{cc} x^{\left(1\right)} & =\underset{x\in X}{\arg\min}\;\Psi \left(x;J_{\mathrm{fast}}\right)\\ x^{*} & =\underset{x\in X}{\arg\min}\;\Psi \left(x;J_{\mathrm{robust}}\right) \end{array}$$

## 5. Experimental Validation

This section reports hard sample mining results for a four-channel MV detector on a SoftSim–SoftGNSS chain, together with a USRP loopback replay of selected schedules at one traction frequency. Section 5.1 defines both chains; Section 5.2, Section 5.3 and Section 5.4 report SoftSim–SoftGNSS results; Section 5.5 summarizes the wired loopback checks.

### 5.1. Measurement Setup

#### 5.1.1. Digital Baseband Chain

The primary instrumented chain uses SoftSim [30] to generate GPS L1 intermediate-frequency (IF) samples and SoftGNSS [31] for acquisition, tracking, and detector feature extraction. Sampling is 2 MHz (8-bit complex baseband); tracking uses 1 ms coherent integration with DLL/PLL noise bandwidths 2/25 Hz. The first 5 s establish authentic tracking at $C/N_{0}=55$ dB-Hz; thereafter the simulator injects a spoofing component whose code phase lags the authentic signal by 2 chips. Decision variables set power, code, and carrier schedules; SoftGNSS returns $C/N_{0}$, Ratio, Delta, and ELP series, from which four-channel MV statistics and hardness scores $P_{d,\mathrm{upper}}$ are formed. Non-overlapping MV windows of length w=100 ms and the fixed thresholds in Table 1 are used throughout. All cross-frequency comparisons, leave-one-frequency-out (LOTO) folds, and ablations use this chain under matched mining budgets (Table 1), consistent with prior software-receiver evaluations of spoofing detectors [2,3].

#### 5.1.2. Wired Loopback Chain

To place the hardness score under an RF front-end—without re-running the full mining sweep on hardware—selected schedules are replayed as follows:

1. SoftSim exports the complex baseband/IF signal for a fixed schedule x (Baseline, Zero-Init, and PBMO).
2. A USRP X310 upconverts and transmits on a wired loopback.
3. The same X310 digitizes the looped signal; samples are ingested by SoftGNSS with matched sampling rate and center frequency.
4. SoftGNSS recomputes tracking discriminators, four-channel MV traces, and $P_{d,\mathrm{upper}}$ under the same detector thresholds as the digital chain.

The scope of RF replay is in the primary case $f_{t}=0.0575$ Hz with Baseline, Zero-Init, and PBMO. *R* independent captures per schedule for conformal scoring.

The experiments address four questions on the digital chain—hard-sample mining efficiency, generalization (LOTO), module necessity (ablations), and channel interpretability—plus whether RF replay preserves Baseline–PBMO hardness ordering. Settings appear in Table 1.

One SoftSim–SoftGNSS call counts as one evaluation. Offline costs in Table 1 are paid once ($9\times (B_{1}+R)=1845$ archive calls, which also form the n=45 conformal pool, plus 1980 sensitivity-grid calls for the PBMO prior); the online cap $B_{2}=80$ is shared by Zero-Init, kNN-Init, Mean-Traj, and PBMO at each test frequency, whereas Baseline is the unoptimized template scored only by the R=5 conformal protocol. The settings $\alpha_{\mathrm{cp}}=0.15$ (finite-sample level ℓ≈0.889 at n=45), γ, and $\eta_{F}$/$\eta_{P}$ are held fixed across $F_{\mathrm{test}}$; γ and the step sizes map the channel bias into ΔP (dB) and ΔF (Hz), and the channel weights are ratios from $\bar{S}$. Mean ± SD and 95% CIs in the result tables are taken across the six test frequencies (or nine LOTO folds).

### 5.2. Cross-Frequency Main Comparison

The main comparison pits PBMO against an unoptimized Baseline and three initialization schemes on $F_{\mathrm{test}}$, which covers trained, interpolated, and extrapolated frequencies. Baseline applies the frequency-difference-lock strategy from Section 3 without search. As noted above, search methods share $B_{2}=80$; Baseline is the unoptimized analytical template.

Baseline [7]. Also known as the frequency-difference-lock strategy, where both power and frequency residuals are set to zero; $T_{\mathrm{dyn}}$ follows Equation (2). No PS or warm start is used—only conformal re-evaluation—so Baseline $P_{d,\mathrm{upper}}$ marks the reference hardness level for the analytical frequency-difference-lock strategy without mining.

Zero-Init. Search starts from an all-zero vector with bounded Gaussian perturbations on power, frequency, and temporal proportions, then projects onto X. No training-archive information is used.

kNN-Init. The training frequency nearest $f_{t}^{\mathrm{new}}$ supplies $x_{\mathrm{nn}}^{*}$, which is projected onto X. Similarity is judged by frequency distance only—no ranking scores or four-channel MV response vectors.

Mean-Traj [12]. Training optima ${\left\{x_{i}^{*}\right\}}_{i=1}^{N}$ are averaged dimension-wise and projected, representing unweighted historical pooling.

Table 2 reports final hardness score $P_{d,\mathrm{upper}}$ by frequency category, together with the mean, sample standard deviation, and Student *t* 95% confidence interval across the six test frequencies. PBMO obtains 0.655, 0.664, and 0.626 on the training, interpolation, and extrapolation subsets, and 0.648±0.035 overall. Relative to the unoptimized frequency-difference-lock strategy (Baseline, 0.935±0.006), this is a 30.7% reduction of the mean. Under the shared online cap $B_{2}=80$, the mean is 24.9% below Zero-Init (0.863±0.094), 14.2% below kNN-Init (0.755±0.058), and 19.2% below Mean-Traj (0.802±0.053). Paired across the same six frequencies, the mean reductions vs. Zero-Init, kNN-Init, and Mean-Traj are 0.215, 0.107 and 0.154. kNN-Init is the strongest equal-budget comparison; Mean-Traj can underperform Zero-Init at hull boundaries where averaging blurs distant training trajectories.

**Figure 4:**
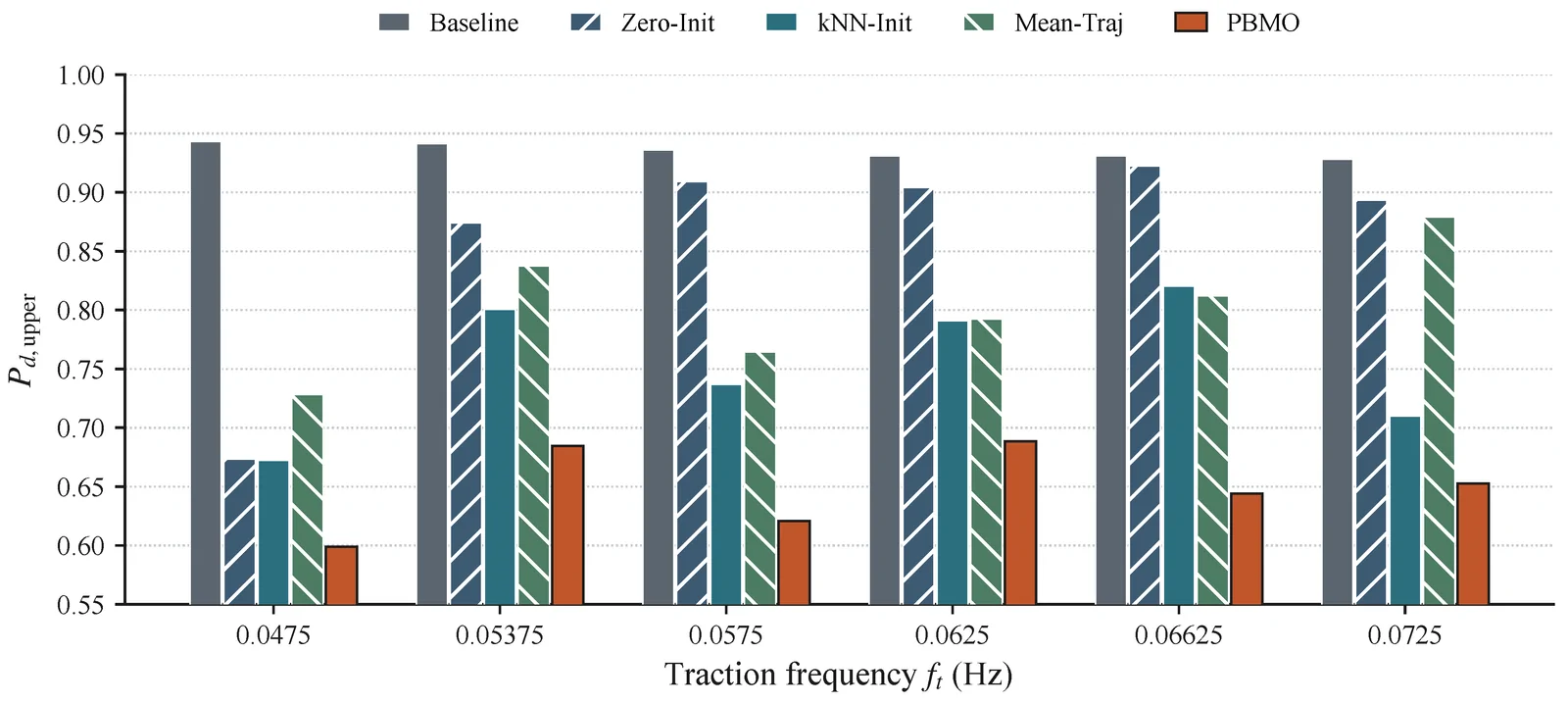
Per-frequency comparison of hardness score $P_{d,\mathrm{upper}}$ across test frequencies.

Figure 4 breaks down results by test frequency:

(1) Low-frequency extrapolation (0.0475 Hz). Zero-Init and kNN-Init already beat Baseline (0.673/0.672 vs. 0.943), whereas Mean-Traj remains elevated (0.728). Mechanistically, this frequency lies outside the lower bound of $F_{\mathrm{train}}$; direct mean pooling causes the ΔF window sequence to mismatch with the MV channel, resulting in an elevated final result. The proposed warm start (0.838) with PhGPS refinement reaches 0.599—showing channel-guided polling is particularly critical for extrapolation points.

(2) High-frequency extrapolation (0.0725 Hz). Cold start is hardest here: $T_{\mathrm{dyn}}$ is short and in-window frequency changes are dense, so Zero-Init (0.894) nears Baseline (0.928). kNN-Init (0.710) still helps; Mean-Traj (0.879) does not. PBMO reaches 0.653, the best single-frequency score. Rising $\rho_{F}$ with $f_{t}$ in Figure 3 suggests stronger frequency-channel coupling that physics-based MV-channel priors exploit.

(3) Interpolation (0.05375, 0.06625 Hz). Zero-Init scores are among the worst (0.874, 0.922) because coordinate PS from scratch wastes budget on weakly coupled dimensions. kNN and mean pooling remain unstable at 0.05375 Hz. PBMO delivers the largest interpolation gain at 0.06625 Hz (30.2%) and wins every interpolation pairing against Zero.

(4) Training-grid frequencies (0.0575, 0.0625 Hz). Headroom shrinks relative to extrapolation. At 0.0575 Hz, kNN-Init reaches 0.737, yet PBMO improves to 0.621 through neighbor blending and channel correction. At 0.0625 Hz, PBMO (0.688) beats kNN-Init (0.791).

### 5.3. LOTO Validation

**Figure 5:**
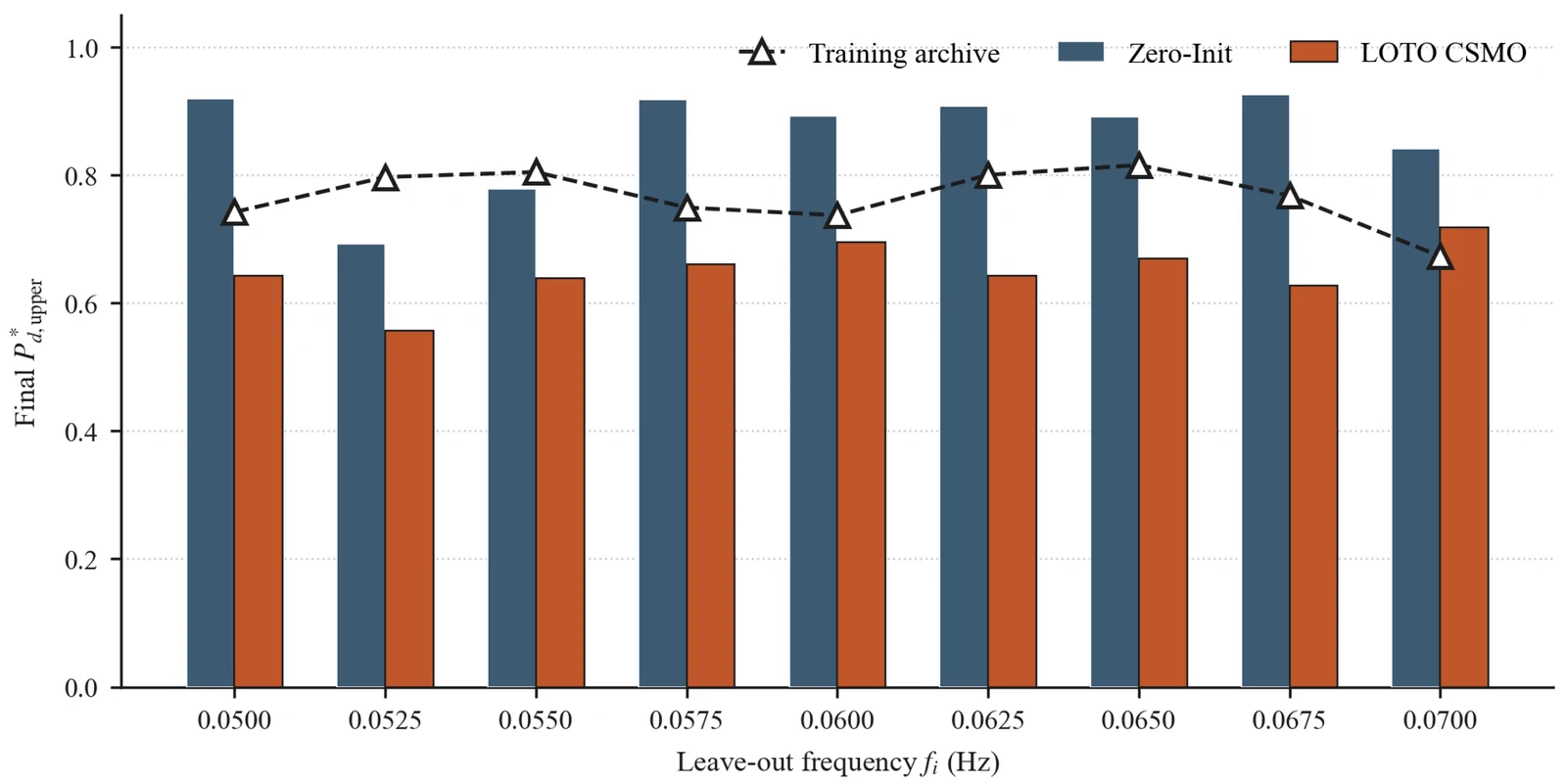
LOTO validation: Zero-Init vs. PBMO final $P_{d,\mathrm{upper}}$ and optimal $P_{d,\mathrm{upper}}$ from the original training archive (dashed reference).

LOTO on $F_{\mathrm{train}}$ tests whether PBMO generalizes rather than memorizes training labels. Each fold holds out one training frequency $f_{i}$, rebuilds warm-start archives from the remaining eight, and compares PBMO with Zero-Init on $f_{i}$ (Table 3, Figure 5).

As shown in Table 3, the nine-fold mean final $P_{d,\mathrm{upper}}^{*}$ is 0.650±0.046 (95% CI [0.615,0.685]) for PBMO vs. 0.863±0.079 ([0.802,0.924]) for Zero-Init (Gain 24.3±6.0%). PBMO wins on every held-out frequency.

At the upper boundary 0.0700 Hz, LOTO $P_{d,\mathrm{upper}}^{*}$ (0.718) is worse than the training-archive optimum (0.673), so the held-out fold cannot be explained by replaying the stored label alone; Gains over Zero-Init nonetheless remain large across the grid. The largest gains appear at hull edges, where cross-frequency blending is most valuable.

### 5.4. Component Ablation

#### 5.4.1. Warm-Start Module Ablation

Warm-start ablations remove Offline-RGPE or physics-based MV-channel priors while keeping all else fixed. *w*/*o* RGPE replaces the RGPE skeleton with Zero-Init but still applies physics-based MV-channel correction from an authentic v evaluation; *w*/*o* MV-prior uses $x_{\mathrm{rgpe}}$ without physics-based MV-channel correction. Table 4 reports the warm-start result.

The full warm-start pipeline averages Init$P_{d,\mathrm{upper}}^{\left(0\right)}$ of 0.786±0.057 vs. 0.912±0.024 without RGPE—a 13.8% relative reduction of the mean—showing that physics-based MV-channel priors alone cannot substitute for a task-relevant strategy skeleton. Removing physics-based MV-channel priors raises Init to 0.812±0.079 (3.3% above the full warm-start pipeline); PBMO beats *w*/*o* MV-prior at every test frequency. The MV prior contributes that 3.3% Init change through a single projected step $\delta_{\mathrm{ch}}$ on $x_{\mathrm{rgpe}}$. Under the same warm start, PhGPS then lowers Init from 0.786±0.057 to 0.648±0.035 (17.2±6.7% relative to Init; Table 5), whereas standard polling reaches 0.762±0.066. The prior, therefore, acts mainly through the poll order in Equation (34).

RGPE contributes most at training frequencies 0.0575/0.0625 Hz (0.742/0.799 vs. 0.905/0.931 without RGPE). Physics-based MV-channel priors help most at 0.05375 Hz, where the RGPE skeleton is already feasible and v-guided ΔP/ΔF tuning suppresses MV exceedance. At 0.0475 Hz, warm-start Init (0.838) exceeds Zero-Init (0.673), but PhGPS refinement still reaches 0.599.

#### 5.4.2. PhGPS Ablation

PhGPS is compared against StdPS under the same Offline-RGPE + channel-prior warm start reported in Table 4, only poll ordering differs.

As shown in Table 5, StdPS only mildly improves the shared Init (0.762±0.066), whereas PhGPS reaches 0.648±0.035—a 17.2±6.7% mean Gain relative to Init. The largest Init-relative gain appears at 0.0475 Hz (28.5%), confirming that channel-aware poll ordering improves local search efficiency when warm start alone is insufficient. Together with Table 4, these figures place most of the reduction from the RGPE skeleton (Init without the MV prior, 0.812±0.079) to the final PBMO score (0.648±0.035) in guided refinement under the shared online cap.

### 5.5. RF Loopback Replay

The comparisons above are entirely digital. Here we ask a narrower question: if a schedule already found in SoftSim–SoftGNSS is played through an RF front-end, does the receiver still pull off, and does the relative detection activation among methods stay roughly the same? We take fixed Baseline, Zero-Init, and PBMO signals from the digital archive, send them through a USRP X310 wired loopback, and reprocess the digitized samples in SoftGNSS (Section 5.1.2).

#### 5.5.1. Replay Protocol and Hardware Configuration

SoftSim writes the complex IF file for each schedule. The X310 upconverts, drives an attenuated cable loopback, and digitizes the return. SoftGNSS then tracks the signal and recomputes the same MV detectors and $P_{d,\mathrm{upper}}$ as on the digital chain. Table 6 gives the front-end settings used for the captures below.

#### 5.5.2. MV Outputs Under RF Loopback

**Figure 6:**
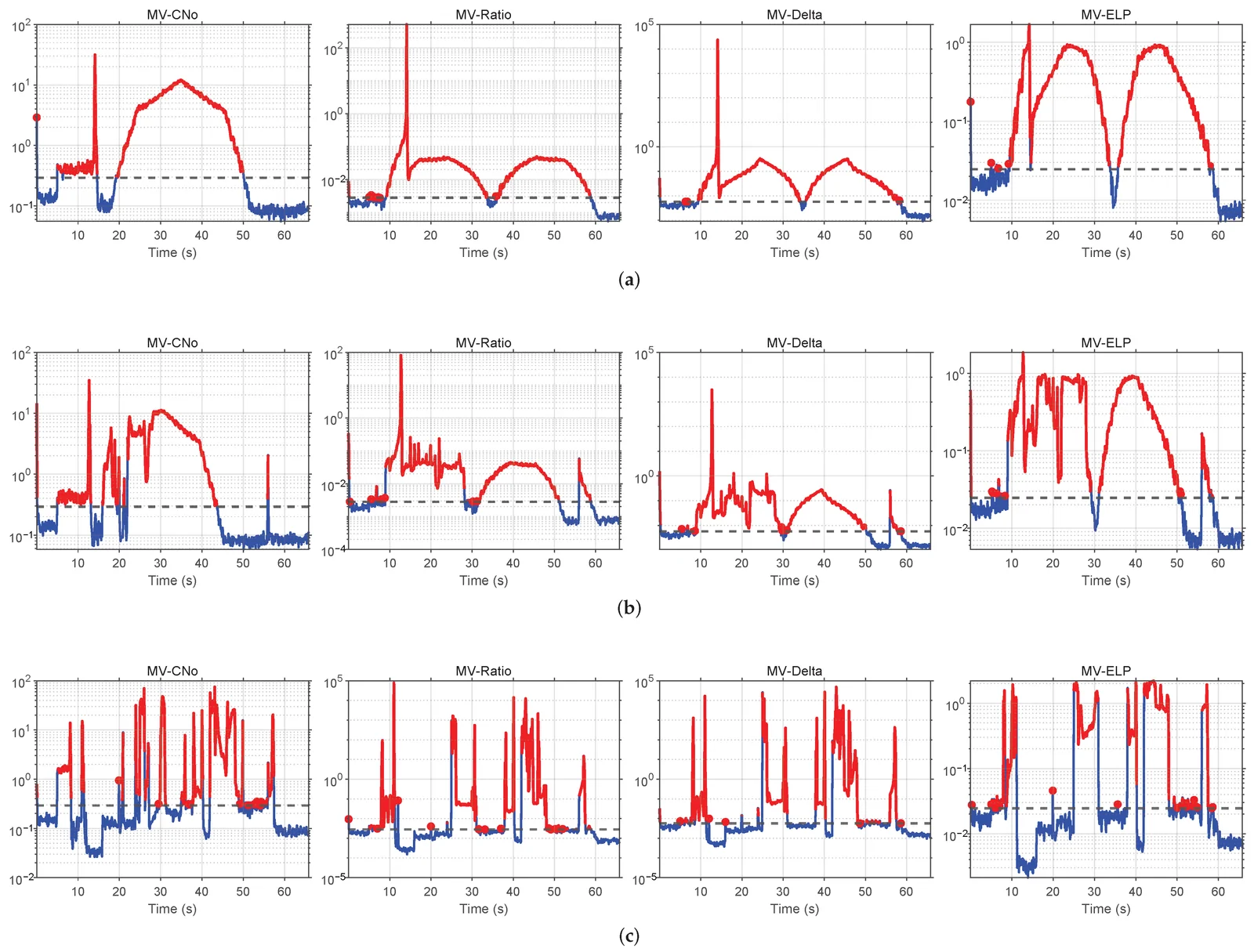
RF loopback: four-channel MV outputs (blue: below threshold; red: above threshold; black dashed: channel detection threshold) at $f_{t}=0.0575$ Hz. (**a**) Baseline; (**b**) Zero-Init; (**c**) PBMO.

Figure 6 shows four-channel MV traces at $f_{t}=0.0575$ Hz after the loopback. Under the PBMO mined hard sample, the four MV channels show lower early detection activation; Baseline and Zero-Init remain above threshold longer, indicating easier early-stage detection under those reference schedules. Absolute MV levels differ slightly from the digital chain—as expected with 8-bit IQ and converter noise—but the same hardness ranking is visible by eye. All three schedules still achieve the commanded Doppler pull-off on the RF path.

#### 5.5.3. Digital vs. RF Hardness Scores

Table 7 puts numbers on that comparison. At $f_{t}=0.0575$ Hz the digital hardness scores are 0.621/0.909/0.936 for PBMO, Zero-Init, and Baseline; after the X310 loopback they become 0.632/0.914/0.943. Every method is a little higher on the RF path, but the hardness order does not change, and PBMO keeps a large gap to the other two.

## 6. Discussion

We interpret the hard sample mining results in four parts: mining efficiency on the digital chain, RF loopback repeatability, cross-frequency generalization, and module roles.

Hard-sample mining efficiency. Against the unoptimized frequency-difference-lock strategy, PBMO lowers mean $P_{d,\mathrm{upper}}$ from 0.935±0.006 to 0.648±0.035, a 30.7% reduction of the mean. Under the shared online cap $B_{2}=80$, the same mean is 24.9% below Zero-Init and 14.2% below kNN-Init; paired mean reductions across the six test frequencies are 0.215 and 0.107 (95% CI [0.133,0.296] and [0.063,0.150]). The archive and sensitivity-grid costs in Table 1 are paid once and excluded from $B_{2}$; kNN-Init and Mean-Traj share the archive, while PBMO alone uses the sensitivity grid. Lower $P_{d,\mathrm{upper}}$ under the pull-off constraint marks a harder sample for this MV detector, so the equal-budget gaps measure hard-sample mining efficiency inside the online cap.

RF loopback. On the X310 wired replay at $f_{t}=0.0575$ Hz (Section 5.5), all three schedules still pull off, and the MV time series keep the same qualitative shape as in SoftSim–SoftGNSS: lower early activation under PBMO, a mid-maneuver peak, then decay. Front-end noise lifts $P_{d,\mathrm{upper}}$ slightly for every method, but PBMO stays well below Zero-Init and Baseline, so the digital hardness ranking is preserved after RF conversion. The check is a conducted cable loopback at one frequency and includes converter noise.

Generalization. LOTO beats Zero-Init on all nine held-out frequencies (0.650±0.046 vs. 0.863±0.079). A two-sided paired Wilcoxon signed-rank test on these nine frequency pairs gives $p=2\times 10^{-3}$. The pairs are held-out traction targets under the fixed protocol; the reported SD and CI describe cross-fold dispersion of the conformal scores. At 0.0700 Hz the LOTO score is worse than the training-archive optimum, so PBMO cannot be explained by label replay alone.

Module roles. Offline-RGPE drives warm-start quality. The MV prior changes the warm-start score by 3.3% (Table 4) and, through poll ordering, accounts for most of the further drop to the final score (Table 5: PhGPS 0.648±0.035 vs. standard polling 0.762±0.066).

Scope and outlook. The demonstrated setting is a hardness ordering for the four-channel MV detector on the digital chain, with a one-frequency wired loopback that preserves the order. The hardness score is a marginal conformal bound on the window-exceedance fraction at nominal level 0.85, under the exchangeability assumption in Section 3.3.2. Receiver-side SoftGNSS variability enters each score through R=5 conformal replicates; table SD and CI summarize dispersion across frequencies, or LOTO folds under that protocol. Physics-based MV-channel priors are tied to the fixed thresholds of this monitor. Natural next steps are curriculum use of the mined trajectories [6], attachment of the miner to other monitors with their own response vectors and sensitivity matrices [5,21], and radiated RF evaluation.

## 7. Conclusions

We have presented Physics-Based Meta-Learning Optimization (PBMO) as a hard sample miner for a GNSS spoofing detector under asynchronous traction stimuli. The hardness score is a conformal upper bound on the MV window-exceedance fraction at a marginal finite-sample level of 0.85 under the exchangeability assumption of Section 3.3.2. PBMO mines hard trajectories through an offline ranking-weighted blend of archived trajectories and physics-based priors, with PhGPS refinement under a fixed online SoftGNSS budget. On the four-channel MV detector, the mean score is 0.648, 30.7% lower than the unoptimized frequency-difference-lock strategy and lower than cold-start, nearest-neighbor, and mean-trajectory search at the same online cap. The archive and sensitivity-grid costs are reported separately. A USRP X310 wired loopback at one traction frequency preserves the hardness order. Curriculum learning and other detectors remain prospective uses of this mining workflow. 

## Figures and Tables

**Table 1:** Experimental parameter settings.

Category	Parameter	Value
Number of time windows	*K*	20
Decision dimension	*n*	59
Traction dynamic duration	$T_{\mathrm{dyn}}$ (s)	$3.5/f_{t}$
Feasibility threshold	τ (${\mathrm{Hz}}^{2}$)	$10^{-4}$
Search bounds	ΔP/ΔF	[−4,4] dB/$\left[-f_{t},f_{t}\right]$ Hz
Baseline spoofing advantage	—	4 dB
MV window/thresholds	*w*; $T_{C},T_{R},T_{D},T_{E}$	100 ms; 0.2916, 0.0028, 0.0057, 0.0246
Training frequencies	$F_{\mathrm{train}}$ (Hz)	{0.050, 0.0525, 0.055, 0.0575, 0.060, 0.0625, 0.065, 0.0675, 0.070}
Multi-start count	$n_{\mathrm{start}}$	8
PS budget per scenario	$B_{1}$	200
Multi-replicate re-evaluation	*R*	5
Conformal significance level	$\alpha_{\mathrm{cp}}$	0.15
Offline archive/sensitivity calls	—	1845/1980
RGPE ranking temperature	$\tau_{r}$	0.04
MV-channel prior gain	γ	0.35
Channel correction step size	$\eta_{F}$/$\eta_{P}$	0.006/0.12
Channel weights	$w_{R}^{F}$/$w_{D}^{F}$/$w_{R}^{P}$	0.92/0.87/0.48
Test frequencies	$F_{\mathrm{test}}$ (Hz)	{0.0475, 0.05375, 0.0575, 0.0625, 0.06625, 0.0725}
Online evaluation budget	$B_{2}$	80
Conformal final-evaluation replicates	—	5
Contraction factor	ρ	0.5
Zero-init perturbation std. dev.	$\sigma_{P},\sigma_{F},\sigma_{T}$	0.20/0.01/0.05

**Table 2:** Final hardness score $P_{d,\mathrm{upper}}$ (lower is harder).

Method	Training	Interp.	Extrap.	Mean ± SD	95% CI
Baseline	0.934	0.936	0.936	0.935±0.006	[0.929,0.941]
Zero-Init	0.907	0.898	0.783	0.863±0.094	[0.764,0.962]
kNN-Init	0.764	0.810	0.691	0.755±0.058	[0.694,0.816]
Mean-Traj	0.778	0.825	0.804	0.802±0.053	[0.746,0.858]
**PBMO **	**0.655**	**0.664**	**0.626**	0.648±0.035	[0.612,0.685]

**Table 3:** Final $P_{d,\mathrm{upper}}^{*}$ and Gain on held-out training frequencies under LOTO validation. Mean ± SD and 95% CI are across the nine folds.

$f_{i}$ (Hz)	Zero Final	PBMO Final	Gain (%)
0.0500	0.920	0.643	30.2
0.0525	0.693	0.557	19.6
0.0550	0.779	0.638	18.0
0.0575	0.918	0.661	28.0
0.0600	0.893	0.696	22.1
0.0625	0.908	0.643	29.2
0.0650	0.891	0.670	24.8
0.0675	0.926	0.628	32.2
0.0700	0.841	0.718	14.6
Mean ± SD	0.863±0.079	0.650±0.046	24.3±6.0
95% CI	[0.802,0.924]	[0.615,0.685]	[19.7,28.9]

**Table 4:** Warm-start module ablation: Init$P_{d,\mathrm{upper}}^{\left(0\right)}$. Mean ± SD and 95% CI are across the six test frequencies.

$f_{t}$ (Hz)	PBMO	*w*/*o* RGPE	*w*/*o* MV-prior
0.0475	0.838	0.885	0.864
0.05375	0.864	0.942	0.941
0.0575	0.742	0.905	0.754
0.0625	0.799	0.931	0.805
0.06625	0.759	0.924	0.778
0.0725	0.716	0.884	0.727
Mean ± SD	0.786±0.057	0.912±0.024	0.812±0.079
95% CI	[0.726,0.847]	[0.886,0.937]	[0.729,0.894]

**Table 5:** PhGPS ablation: StdPS vs. PhGPS final $P_{d,\mathrm{upper}}$ (Gain relative to Init). Mean ± SD and 95% CI are across the six test frequencies.

$f_{t}$	Init	StdPS	PhGPS	Gain (%)
0.0475	0.838	0.788	0.599	28.5
0.05375	0.864	0.852	0.684	20.8
0.0575	0.742	0.738	0.621	16.3
0.0625	0.799	0.789	0.688	13.9
0.06625	0.759	0.752	0.644	15.1
0.0725	0.716	0.655	0.653	8.8
Mean ± SD	0.786±0.057	0.762±0.066	0.648±0.035	17.2±6.7
95% CI	[0.726,0.847]	[0.693,0.831]	[0.612,0.685]	[10.2,24.3]

**Table 6:** USRP X310 wired-loopback configuration.

Item	Setting
USRP	X310
TX/RX center frequency	1575.42 MHz
Sample rate	2 MHz
IQ bit	8
SoftGNSS coherent integration	5

**Table 7:** Digital-chain vs. USRP X310 loopback $P_{d,\mathrm{upper}}$ at $f_{t}=0.0575$ Hz.

Method	Digital	X310 Loopback
Baseline	0.936	0.943
Zero-Init	0.909	0.914
**PBMO**	**0.621**	**0.632**

## Data Availability

The data presented in this study are available from the corresponding author upon reasonable request.
